# Supplementary material for: Generation of Chloroplast Molecular Markers to Differentiate Sophora toromiro and Its Hybrids as a First Approach to Its Reintroduction in Rapa Nui (Easter Island)
Source: Plants (Basel). 2021 Feb 10;10(2):342. doi: 10.3390/plants10020342 (PMC7916652; doi:10.3390/plants10020342)
Supplement: Supplementary file 1 [file plants-10-00342-s001.zip › sup_materials/toromiro_supplementary.docx]

**Supplementary information.**

**Figures**


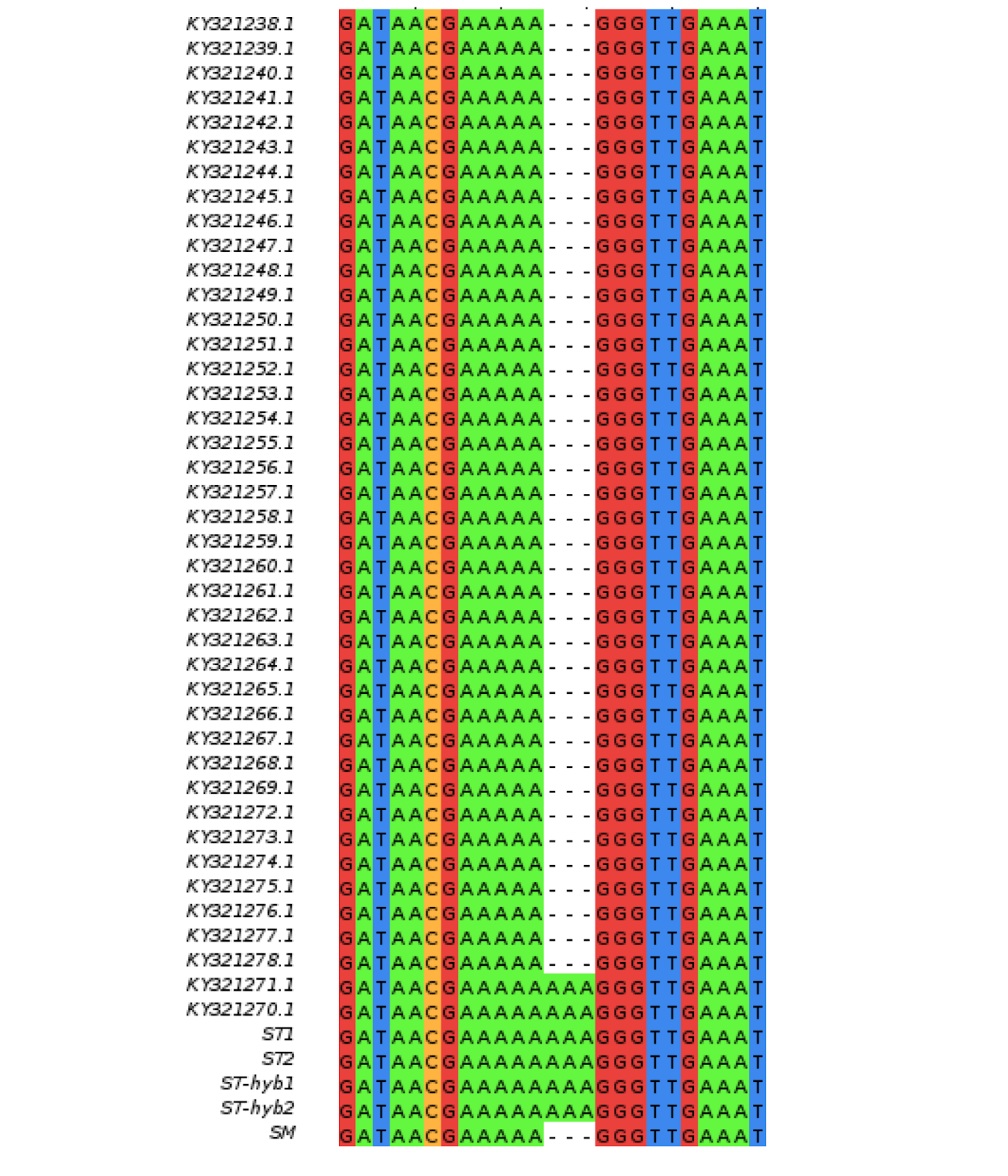


**Supplementary Figure 1.** Multiple sequence alignment of the psbA/trnH intergenic spacer of different *Sophora* species showing the 3 bp insertion (AAA). KY321270.1 and KY321271.1 are *S. toromiro* herbarium samples. *S. cassioides* = KY321261, KY321263, KY321266, KY321267, KY321268; *S. chathamica* = KY321239, KY321241, KY321244, KY321247, KY321257; *S. chrysophylla* = KY321265; *S. denudata* = KY321278; *S. fernandeziana* = KY321274; *S. fulvida* = KY321269; *S. howinsula* = KY321269; *S. macrocarpa* = KY321264, KY321260, KY321262, KY321276; *S. mangarevaensis* = KY321273; *S. microphylla* = KY321245, KY321246, KY321248, KY321250, KY321251, KY321252, KY321253; *S. prostrata* = KY321243, KY321256; *S. raivavaeensis* = KY321277; *S. rapaensis* = KY321275, KY321272; *S. tetraptera* = KY321238, KY321242, KY321249, KY321254, KY321255, KY321258, KY321259.


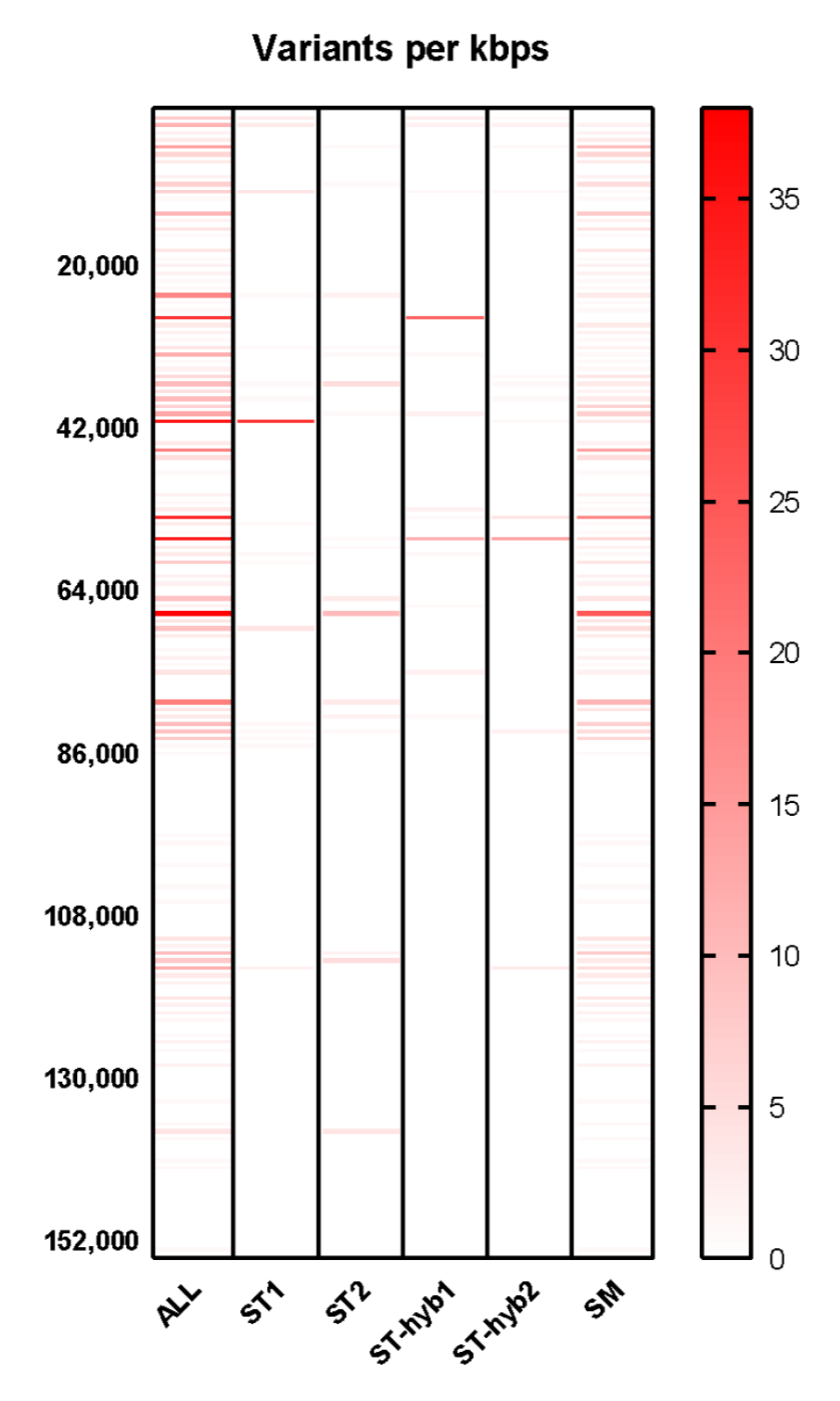


**Supplementary Figure 2.** Variants detected using the full set of 577 SNP with all the samples together (ALL) and unique SNPs for each sample. ST2 annotation data was used as reference for calculating gene variants. Each row represents a window of 1 kbps. Number of variants caused by SNPs are represented with a red coloration to the right.


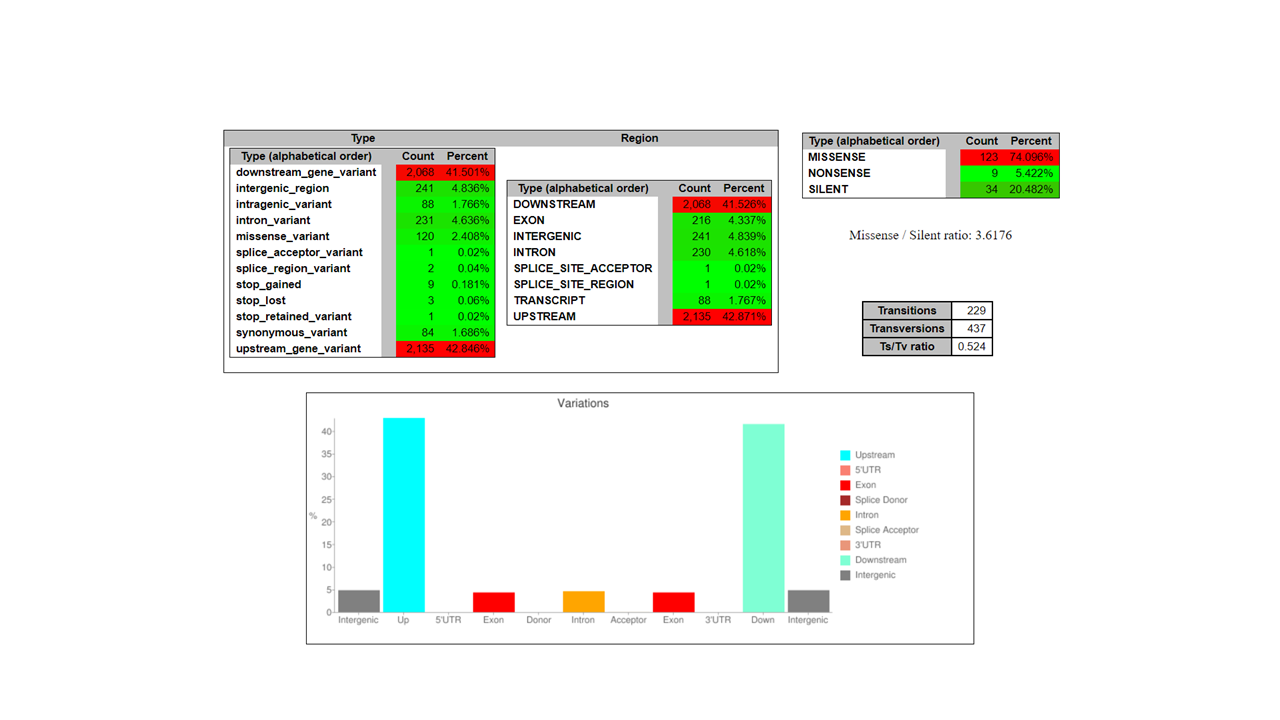


**Supplementary Figure 3.** Number of SNP effects by type and regions. Data obtained from default parameters of SnpEff.


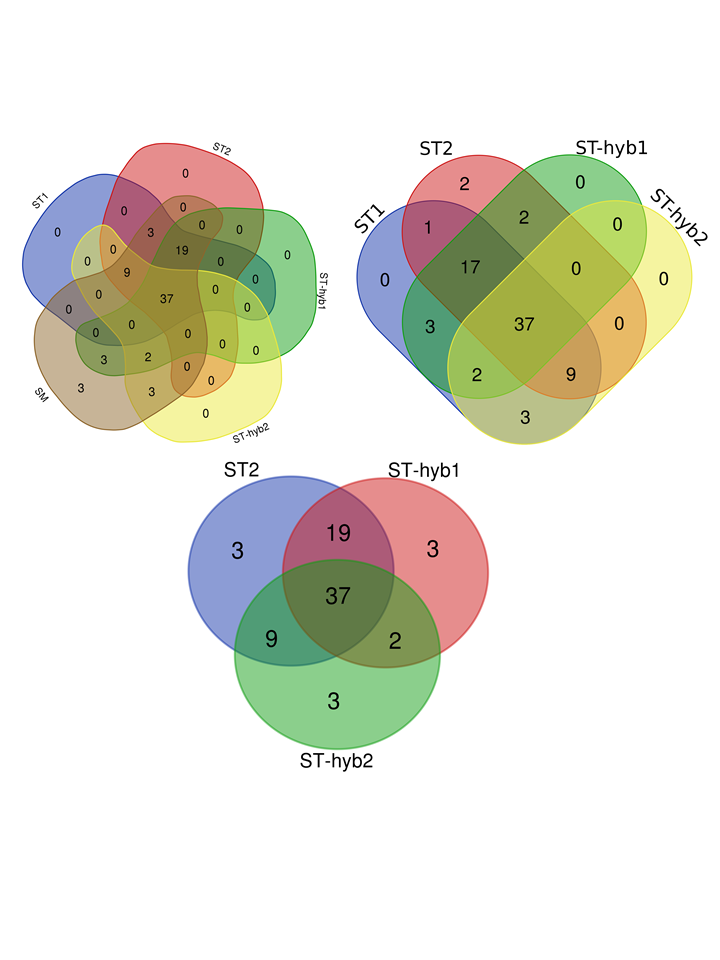


**Supplementary Figure 4.** Venn diagram comparing proteins coding genes with variants between the 5 samples (Upper left), 4 samples (Upper right), and 3 samples (bottom).

**Tables**

**Supplementary Table 1.** Full SSR characterization.

Too big to show here.

**Supplementary Table 2.** Number of SNPs after filtering for SnpEff analysis

| **Sample set** | **N° of SNPs** |
| --- | --- |
| Whole samples | 577 |
| Unique ST1 | 57 |
| Unique ST2 | 44 |
| Unique ST-hyb1 | 52 |
| Unique ST-hyb2 | 32 |
| Unique SM | 303 |
| Shared between 2 samples | 89 |
