# Supplementary figures and images for: Generation of Chloroplast Molecular Markers to Differentiate Sophora toromiro and Its Hybrids as a First Approach to Its Reintroduction in Rapa Nui (Easter Island)
Source: Plants (Basel). 2021 Feb 10;10(2):342. doi: 10.3390/plants10020342 (PMC7916652; doi:10.3390/plants10020342)

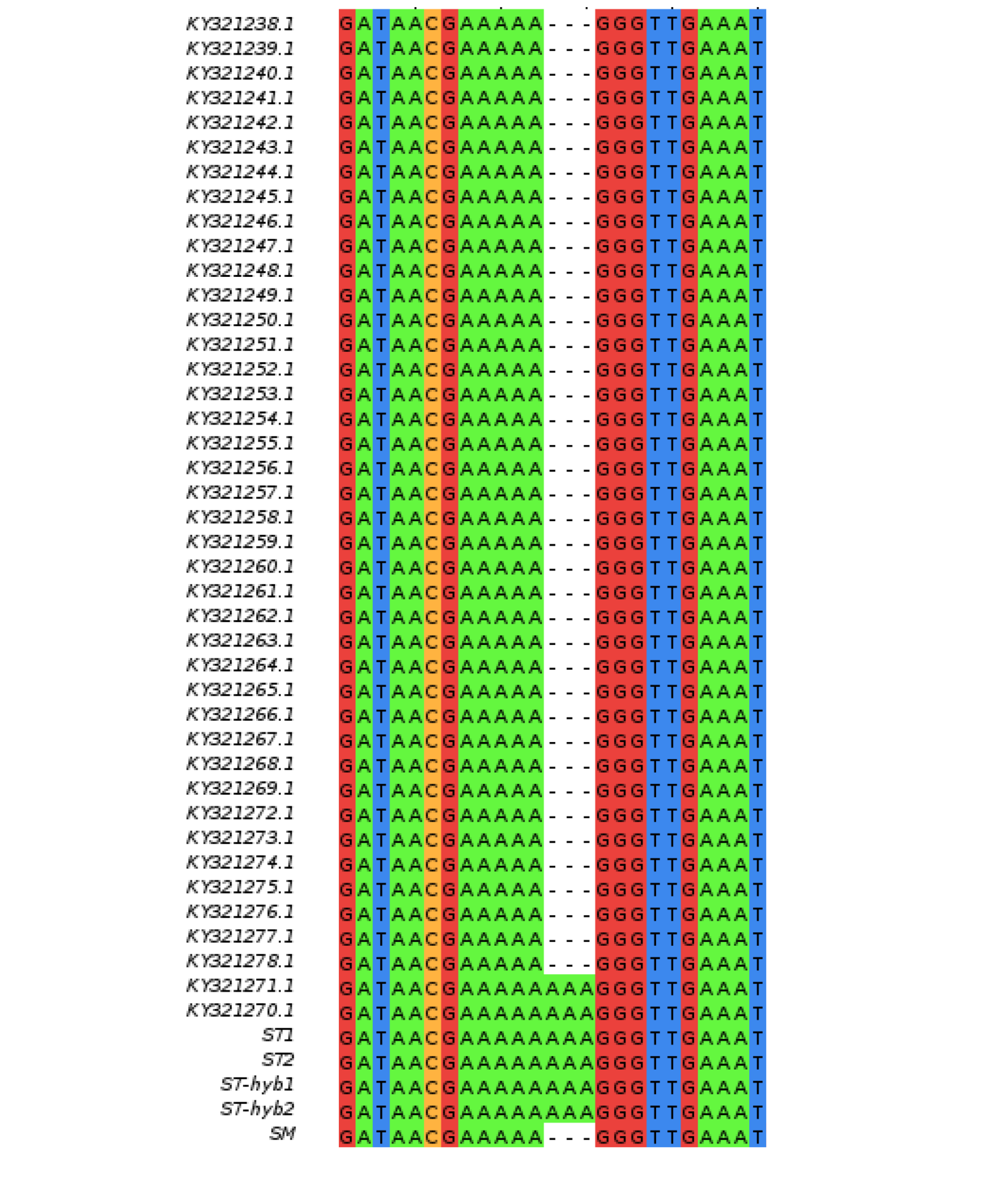

Supplement: Supplementary file 1 [file plants-10-00342-s001.zip › sup_materials/sup_figure1.png]

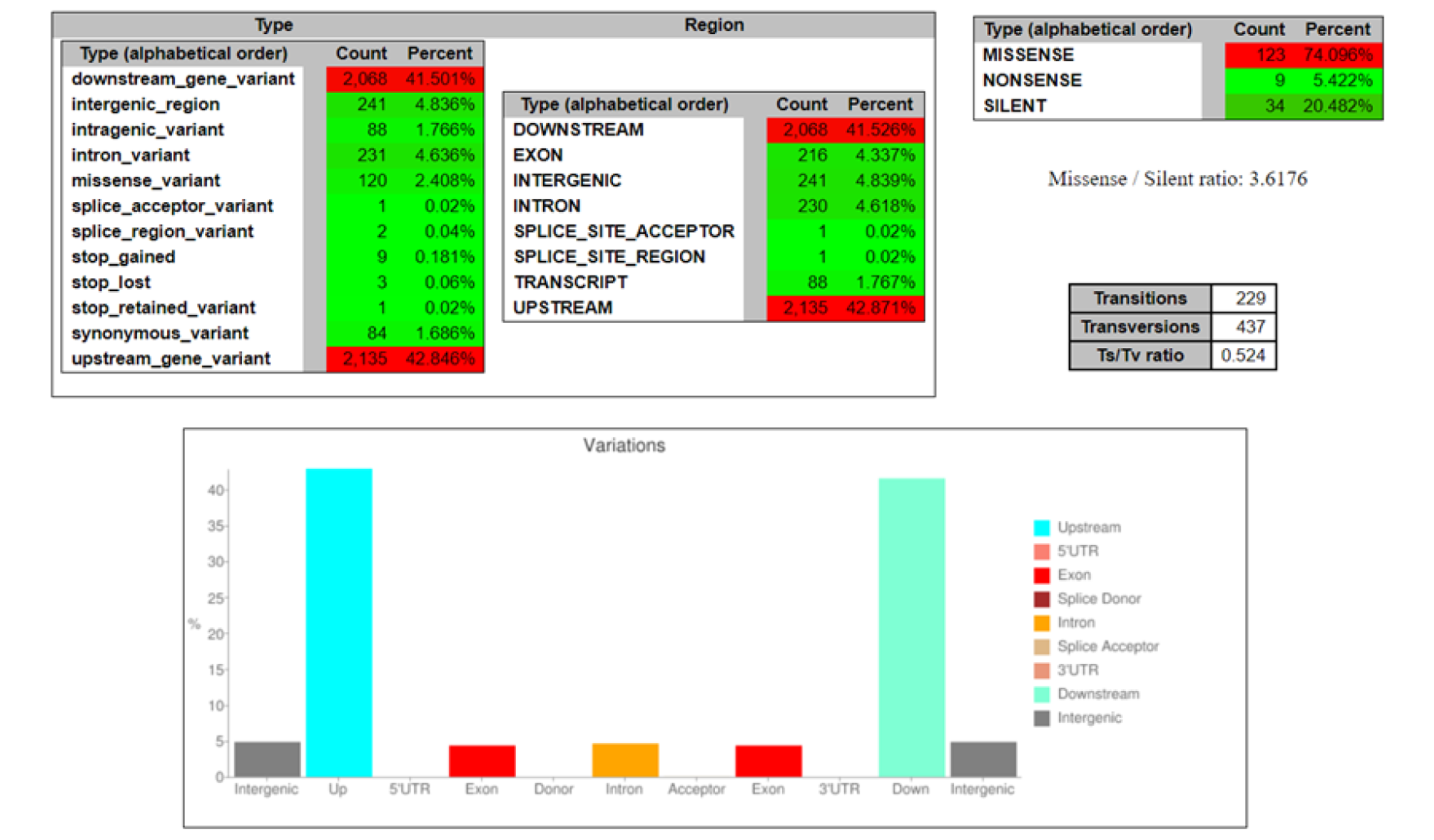

Supplement: Supplementary file 1 [file plants-10-00342-s001.zip › sup_materials/sup_figure3.png]

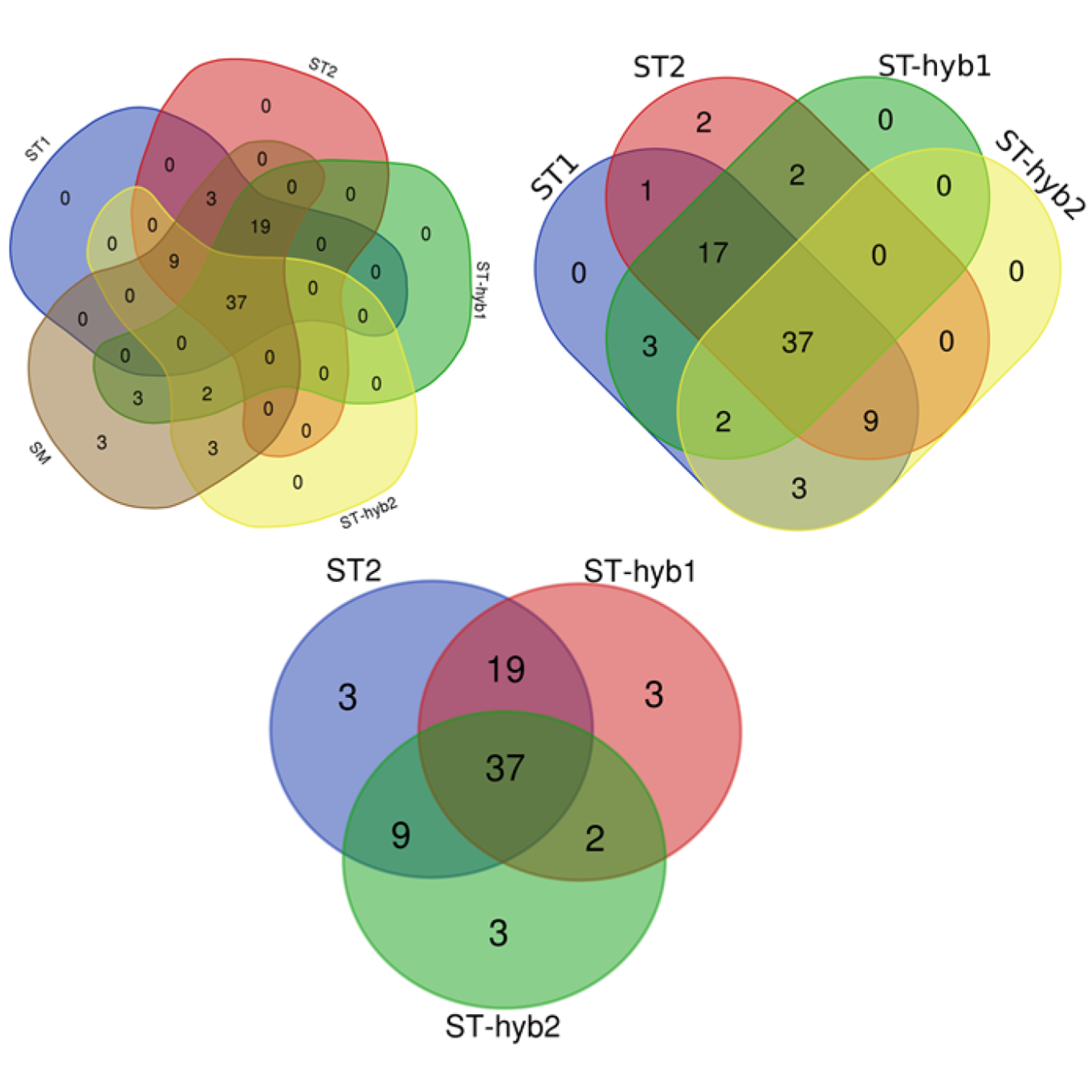

Supplement: Supplementary file 1 [file plants-10-00342-s001.zip › sup_materials/sup_figure4.png]

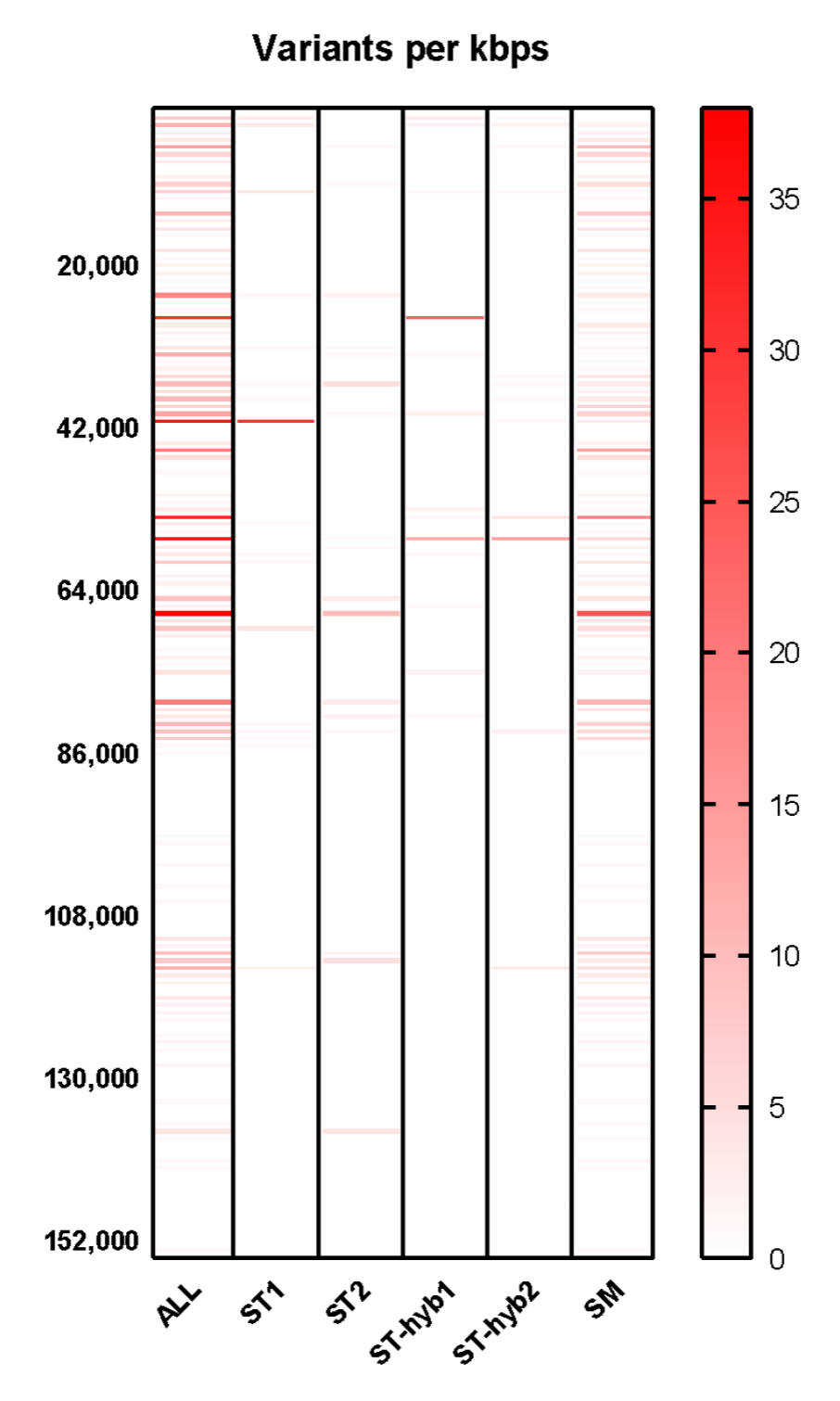

Supplement: Supplementary file 1 [file plants-10-00342-s001.zip › sup_materials/sup_figure2.png]
